# The efficacy of a coordinated pharmacological blockade in glioblastoma stem cells with nine repurposed drugs using the CUSP9 strategy

**DOI:** 10.1007/s00432-019-02920-4

**Published:** 2019-04-26

**Authors:** Erlend Skaga, Ida Ø. Skaga, Zanina Grieg, Cecilie J. Sandberg, Iver A. Langmoen, Einar O. Vik-Mo

**Affiliations:** 1 Vilhelm Magnus Laboratory, Institute for Surgical Research and Department of Neurosurgery, Oslo University Hospital, P.O. Box 4950, Nydalen, 0424 Oslo, Norway; 2 Faculty of Medicine, Institute of Clinical Medicine, University of Oslo, P.O. Box 1112, Blindern, 0317 Oslo, Norway

**Keywords:** Glioblastoma stem cells, CUSP9, Temozolomide, Glioblastoma, Repurposed drugs

## Abstract

**Purpose:**

Constructed from a theoretical framework, the coordinated undermining of survival paths in glioblastoma (GBM) is a combination of nine drugs approved for non-oncological indications (CUSP9; aprepitant, auranofin, captopril, celecoxib, disulfiram, itraconazole, minocycline, quetiapine, and sertraline) combined with temozolomide (TMZ). The availability of these drugs outside of specialized treatment centers has led patients to embark on combination treatments without systematic follow-up. However, no experimental data on efficacy using the CUSP9 strategy in GBM have been reported.

**Methods:**

Using patient-derived glioblastoma stem cell (GSC) cultures from 15 GBM patients, we described stem cell properties of individual cultures, determined the dose–response relationships of the drugs in the CUSP9, and assessed the efficacy the CUSP9 combination with TMZ in concentrations clinically achievable. The efficacy was evaluated by cell viability, cytotoxicity, and sphere-forming assays in both primary and recurrent GSC cultures.

**Results:**

We found that CUSP9 with TMZ induced a combination effect compared to the drugs individually (*p* < 0.0001). Evaluated by cell viability and cytotoxicity, 50% of the GSC cultures displayed a high sensitivity to the drug combination. In clinical plasma concentrations, the effect of the CUSP9 with TMZ was superior to TMZ monotherapy (*p* < 0.001). The Wnt-signaling pathway has been shown important in GSC, and CUSP9 significantly reduces Wnt-activity.

**Conclusions:**

Adding experimental data to the theoretical rationale of CUSP9, our results demonstrate that the CUSP9 treatment strategy can induce a combination effect in both treatment-naïve and pretreated GSC cultures; however, predicting response in individual cultures will require further profiling of GSCs.

**Electronic supplementary material:**

The online version of this article (10.1007/s00432-019-02920-4) contains supplementary material, which is available to authorized users.

## Introduction

Glioblastoma (GBM) is an aggressive brain tumor that, despite multimodal oncological therapy, relapses within 6–9 months (Stupp et al. 2005). A major challenge in developing new treatments is the intricate tumor heterogeneity at the molecular and cellular level (Brennan et al. 2013; Qazi et al. 2017; Lan et al. 2017). Targeted therapies have been sought to address the molecular heterogeneity in GBM, but dozens of clinical trials have failed to demonstrate survival benefit (Touat et al. 2017).

The application of targeted therapies is hampered by the existence of complex intra- and intertumoral heterogeneity in tumor-promoting signaling systems along with tumor evolutionary dynamics leading to acquired resistance to targeted drugs (Szerlip et al. 2012; Sottoriva et al. 2013; Klingler et al. 2015). To circumvent tumor heterogeneity, polytherapeutic approaches combining compounds acting on different targets simultaneously are receiving rising interest (Qazi et al. 2017). Moreover, the dramatic increase in costs for new oncological drugs has increased both the academic and public interest into possibilities in repurposing well-known drugs used for non-oncological indications for their potential anticancer activity (Bertolini et al. 2015; Huang et al. 2018). And as drugs used for decades have established dosing schedules and well-known toxicity profiles, both the time frame and costs to reposition for new indications can greatly be reduced.

Recently, a new treatment approach combining well-known drugs approved for non-oncological indications for polytherapeutic therapy has been suggested in GBM (Kast et al. 2013, 2014). The rationale consists of coordinated undermining of survival paths (CUSP) active in GBM by nine repurposed drugs, termed CUSP9. The concept of simultaneous blockade of multiple signaling pathways aims to prevent cancer cells to escape therapeutic challenges, rendering them susceptible for the cytotoxic effects of temozolomide (TMZ). Due to toxicities, the composition of drugs has been revised, and the current version consists of aprepitant, auranofin, captopril, celecoxib, disulfiram, itraconazole, minocycline, quetiapine, and sertraline (Halatsch et al. 2017).

Since the proposal, the concept of CUSP has been debated among neuro-oncology academics and practitioners (Prados et al. 2015; Purow 2016). More importantly, however, we experience that patients inquire and also adhere to parts or the entire CUSP9 combination along standard-of-care treatments outside of clinical trials within a do-it-yourself approach. Although case reports of patients treated with CUSP9 on a compassionate-use basis (Kast et al. 2014; Halatsch et al. 2017), and a recent registration of a clinical trial (NCT02770378), no experimental data have shown efficacy using the CUSP9 strategy. This prompted us to explore the efficacy of CUSP9 with concomitant TMZ using clinical achievable drug concentrations in patient-derived glioblastoma stem cells (GSCs), which may be responsible for tumor progression and recurrence in GBM (Lan et al. 2017).

## Materials and methods

### Brain tumor cultures

Glioblastoma biopsies were obtained from 15 informed and consenting patients undergoing surgery for GBM at Oslo University Hospital, Norway, approved by The Norwegian Regional Committee for Medical Research Ethics (REK 2017/167). The IDH mutational status was evaluated by immunohistochemistry and sequencing, and the MGMT-promoter methylation status evaluated by methylation-specific quantitative PCR. Cell cultures were established and maintained in serum-free conditions enriched for bFGF (R&D Systems) and EGF (R&D Systems), as previously described (Vik-Mo et al. 2010). Patient- and GSC culture characteristics are summarized in Online Resource 1. The self-renewal potential of the GSCs was quantified by the total number of cells following serial passages. Differentiation was induced, and cells were fixed and stained, as previously described (Vik-Mo et al. 2010). Images were acquired using Olympus Soft Imaging Xcellence software v.1.1.

### Flow cytometry

Cells were suspended in PBS with 2% fetal bovine serum (Biochrom) and stained with directly conjugated antibodies (CD15-PerCP, R&D Systems, CD133-PE, Miltenyi Biotec) according to the manufacturer’s instructions. Cells were washed three times before analysis by flow cytometer LSRII (BD Bioscience). Dead cells were identified by propidium iodine (Thermo Fisher Scientific). Flow Jo software v.10.4.1 was used for data analysis.

### qRT-PCR

The qRT-PCR experiments were performed, as previously described (Fayzullin et al. 2019). The high-capacity cDNA Reverse Transcription Kit, TaqMan Fast Advanced Master Mix, TaqMan oligonucleotide primers and probes [Hs00157674_m1 (GFAP), Hs00801390_s1 (TUBB3), and Hs01009250_m1 (PROM1/CD133)], the ABI Prism Detection System, and software (all from Applied Biosystems) were used according to the manufacturer’s instructions. Human β-Actin [Hs9999999903_m1 (ACTB)] was used as housekeeping gene. The relative gene expression levels were calculated using the standard curve method.

### Intracranial transplantation

The National Animal Research Authority approved the animal procedures (FOTS 8318). C.B.-17 SCID female mice (7–9 weeks old, Taconic) were anesthetized with an injection of zolazepam (3.3 mg/mL), tiletamine (3.3 mg/mL), xylazine (0.45 mg/mL), and fentanyl (2.6 μg/mL), and placed in a stereotactic frame (David Kopf Instruments). Cells were prepared and transplanted, as previously described (Vik-Mo et al. 2010). The animals were regularly monitored for signs of distress and killed by cervical dislocation after 15 weeks or earlier if weight loss > 15% or neurological symptoms developed. The brains were harvested and further processed as previously described (Vik-Mo et al. 2010). Images of brain sections were acquired using Axio Scan.Z1 (Carl Zeiss). Processing of images was performed using ImageJ 2.0.

### Drugs

Drugs used in this study: aprepitant (Selleck Chemicals, Cat# S1189), auranofin (Santa Cruz Biotechnology, Cat #sc-202476), captopril (Selleck Chemicals, Cat# S2051), celecoxib (Selleck Chemicals, Cat# S1261), copper(II)chloride dehydrate (Sigma-Aldrich, Cat# C3279), disulfiram (Selleck Chemicals, Cat# S1680), itraconazole (Selleck Chemicals, Cat# S2476), minocycline (Selleck Chemicals, Cat# S4226), quetiapine fumarate (Selleck Chemicals, Cat# S1763), sertraline (Selleck Chemicals, Cat# S4052), and temozolomide (Sigma-Aldrich, Cat# T2577). Copper(II)chloride dehydrate (CuCl_2_) was added to all wells containing disulfiram (DSF) and corresponding control wells (Skrott et al. 2017). A fixed concentration of 20 µM Cu was used in this study (Twomey et al. 2008). Minocycline was dissolved in H_2_O, while all other drugs were dissolved in DMSO for generation of stock solutions and stored according to the manufacturer’s instructions.

### Drug concentrations

The clinical plasma concentrations of the individual drugs were obtained from reports of pharmacokinetic evaluations [aprepitant (Azuma and Fukase 2013), auranofin (Gottlieb 1982; Furst and Dromgoole 1984), captopril (Kripalani et al. 1980; al-Furaih et al. 1991), celecoxib (Davies et al. 2000), disulfiram (Johansson 1988, 1992), itraconazole (Heykants et al. 1989; Prentice et al. 1994), minocycline (Macdonald 1973; Agwuh 2006), quetiapine (DeVane and Nemeroff 2001; Jaskiw et al. 2004), sertraline (DeVane et al. 2002), and temozolomide (Ostermann et al. 2004)], and from drug labels by the U.S. Food and Drug Administration (http://labels.fda.gov).

### Cell viability and cell cytotoxicity assay

Cells were plated at 5000 cells/well in a 96-well plate (Sarstedt), cultured for 24 h before adding drugs and further incubated for 72 h. Cell viability was assessed using Cell Proliferation Kit II XTT (Roche) solution, incubated for 24 h before absorbance was analyzed on a PerkinElmer EnVision. Cell survival is reported relative to background corrected negative control of the drug. Cell cytotoxicity was assessed using CellTox™ Green Cytotoxicity Assay (Promega) solution, incubated for 15 min before fluorescence was analyzed on a Perkin Elmer EnVision. Measurements were corrected for background fluorescence, and raw data were scaled with reference to positive (sepantronium bromide) and negative control.

### Sphere-forming assay

Cells were plated at 500 cells/well in 96-well plate (Sarstedt), cultured for 24 h before adding drugs and further incubated for 10 days. After 10 days, the spheres were stained using Thiazolyl Blue Tetrazolium Bromide (Sigma-Aldrich) 4 h prior to image acquisition and counting using an automated colony counter (GelCount, Oxford Optronics). Spheres > 30 µM in diameter were included in the final analysis, and results are reported relative to negative control.

### Wnt-pathway activity (luciferase assay)

The GSCs were stably transfected with a luciferase reporter containing a synthetic 7xTCF-responsive promoter (7TFP was a gift from Roel Nusse, Addgene plasmid 24308). The lentiviral Renilla luciferase reporter was used as control (Amsbio, Cat# LVP370). The cells were plated at 20,000 cells/well in a 96-well plate before adding the respective drugs of CUSP9. To boost WNT/β-catenin signaling, 10 mM LiCl was added. The cells were incubated for 24 h before luciferase activity was quantified using the Dual-Glo Luciferase Assay System (Promega) according to the manufacturer’s protocol.

### Statistical considerations

Data analysis and graphic presentation were undertaken using GraphPad Prism 7.0 and Microsoft Excel 14.7.3. Dose–response curves were fitted on the basis of a four-parameter sigmoidal logistic fit function defined by maximal and minimal cell survival, slope, and inflection point (EC_50_). In the curve fitting, the maximal cell survival was fixed to 100%, the minimal cell survival was allowed to float between 0% and 75% and slope between 0 and − 2.5. For drugs not reducing any cell survival, the constraints were removed. Statistical analyses were performed using paired sample *t* test or one-way ANOVA corrected for multiple comparisons using Dunnett’s test, as stated when the analysis was applied. Correlation analysis was undertaken using Spearman correlation coefficient. A *p* value < 0.05 was chosen to represent significance for the statistical analyses.

## Results

### Validation of GSCs

We have extensive experience in culturing and characterization of the GSC population from patient-derived GBM biopsies (Varghese et al. 2008; Vik-Mo et al. 2010; Mughal et al. 2018).

**Fig. 1:**
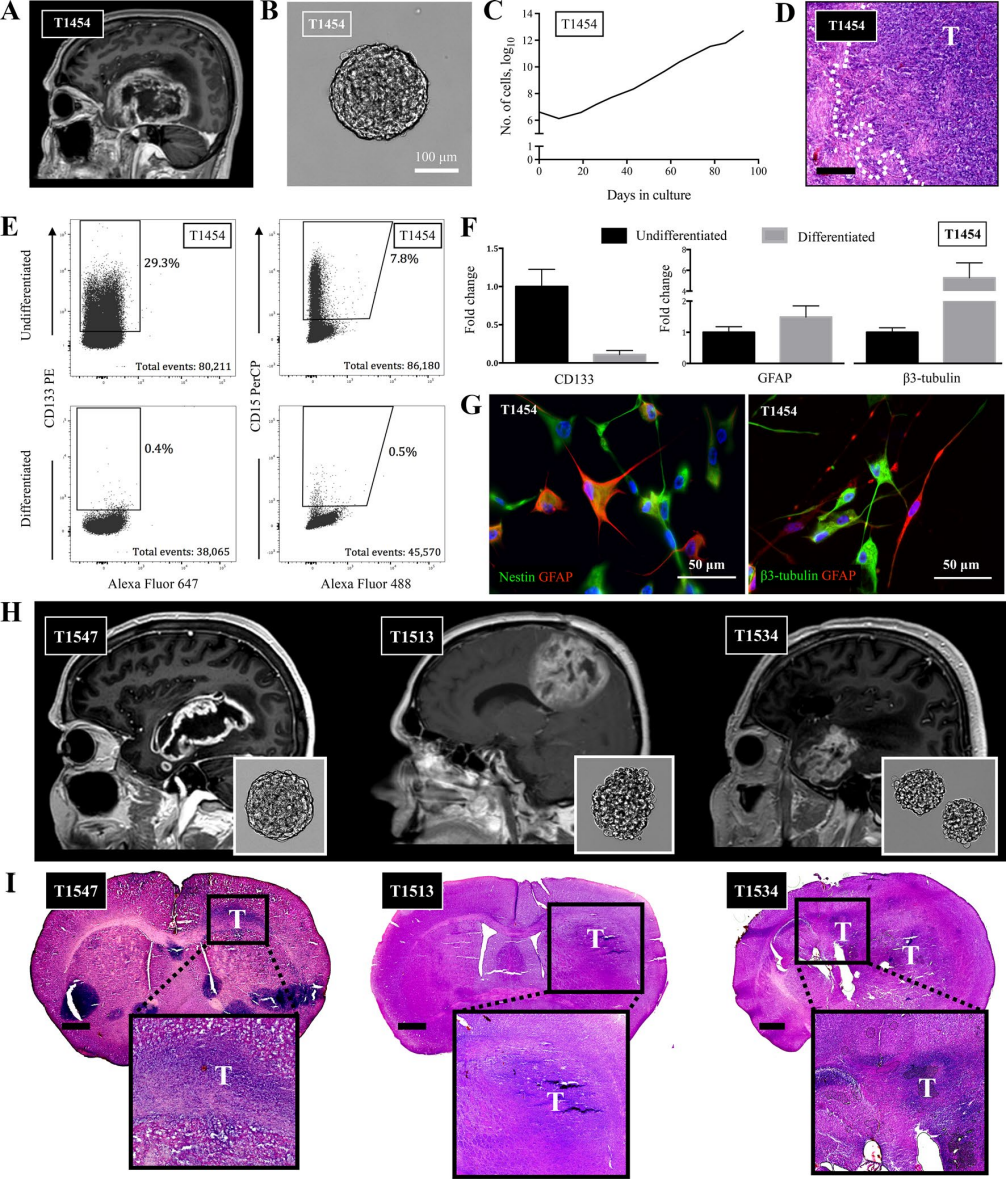
Patient-derived GSCs harbor stem cell properties. **a** T1 contrast-enhanced MRI of T1454 displaying the GBM located in the right temporal region. **b** Upon cultivation, the tumor formed free-floating spheres, **c** which could be exponentially propagated in serial passages. **d** Upon xenografting, the tumor formed an invasive tumor. Invasive rim of the tumor delineated. Staining with hematoxylin and eosin. Scale bar = 200 μm. **e** The GSCs in T1454 expressed the stem cell markers CD133 and CD15. The expression was reduced upon differentiation. **f** qRT-PCR confirmed the reduction of stem cell-related expression of CD133, along with the increased expression of the more lineage-specific GFAP and β3-tubulin upon differentiation. The results are presented as mean and standard error to the mean of three independent experiments. **g** Upon differentiation in serum-containing media, the tumor cells formed arborizations and twisting processes associated with a more mature morphology, and stained positive for GFAP and β3-tubulin. Nuclei stained with DAPI. **h**, **i** T1 contrast-enhanced MRI of the primary GBM T1547 and the pretreated and recurrent GBMs T1513 and T1534 with the corresponding in vitro spheroid morphology upon cultivation and the corresponding xenograft. Brain sections are stained with hematoxylin & eosin. Scale bar 1 mm

We have previously characterized selected GSC cultures (T10965, T1008) in this sample cohort (Vik-Mo et al. 2010; Joel et al. 2015; Mughal et al. 2018). Of the remaining GSC cultures, we confirmed stem cell properties by functional assays of self-renewal potential, expression of stem cell markers, ability to generate different brain cell lineages upon differentiation with differential expression profiles, along with the ability to form tumors upon xenografting to immunocompromised mice in both cultures derived from treatment-naïve and heavily pretreated recurrent disease (Fig. 1, Online Resource 1).

### The dose–response relationships and clinical relative drug concentrations

**Fig. 2:**
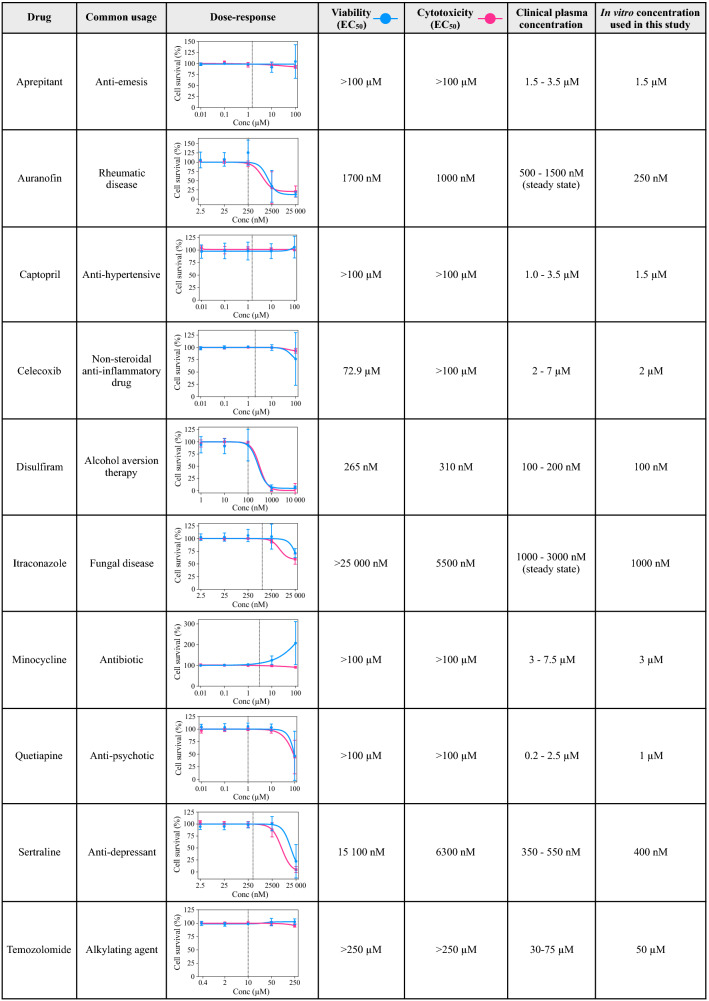
Drugs comprised in the CUSP9 combination, their common usage, dose–response curves, and related drug concentrations used in this study. The dose–response relationships of all the drugs in the CUSP9 combination in GSCs evaluated by cell viability (blue line) and cell cytotoxicity assays (red line). Each dose–response curve represents the average dose–response relationship (± standard deviation) of four different primary GSC cultures (T1456, T1459, T1502, and T1506). Each concentration was tested in biological triplicates in the individual tumor. Half-maximal effective drug concentrations (EC_50_) to the individual drugs, clinical plasma concentrations, and in vitro concentrations used in the CUSP9 combination are provided in the figure along with the common usage of the respective drugs. The vertical line in the dose–response curves represents the in vitro concentration of the individual drug used in this study, whereby the drug individually did not reduce the average cell survival across the GSC cultures

Next, we established the dose–response relationships to all drugs comprised in the CUSP9 and TMZ in four different primary GSC cultures (T1456, T1459, T1502, and T1506, Fig. 2). Each drug was tested in a dose range covering clinically achievable concentrations. To capture both cytostatic (cell viability) and cytotoxic (cell cytotoxicity) responses, we utilized two independent evaluations of cell death. Except for auranofin (AUR) and DSF, no drugs displayed any marked inhibitory effect individually within the concentration range tested (Fig. 2). The marked inhibitory effect (cell survival < 25%) of AUR and DSF was found to be in concentrations well above what could be considered clinically achievable.

After establishing the dose–response relationships, clinical plasma concentrations (CPC) were obtained for each drug. As CPCs vary depending on the dose, route of administration, and drug half-life [maximal concentration (*C*_max_) versus steady-state levels], we decided to pursue drug concentrations in the CUSP9 combination in the lower end of reported *C*_max_-values at standard dosing schedules for the individual drugs intended use (Fig. 2). As AUR and itraconazole (ITZ) have a half-life of > 10 days and > 24 h, respectively (Gottlieb 1982; Heykants et al. 1989; Kast et al. 2014), we used the steady-state drug concentration of AUF and ITZ to reflect a clinical situation. However, as steady-state concentrations of AUR (≥ 500 nM) were within the area of inflection with inhibitory effects in GSC cultures, we used 250 nM of AUR in the CUSP9 combination to remove a possible substantial inhibitory effect of a single drug. The concentrations further pursued in the study related to CPCs are outlined in Fig. 2.

### Combination effect of CUSP9 in patient-derived primary GSC cultures

**Fig. 3:**
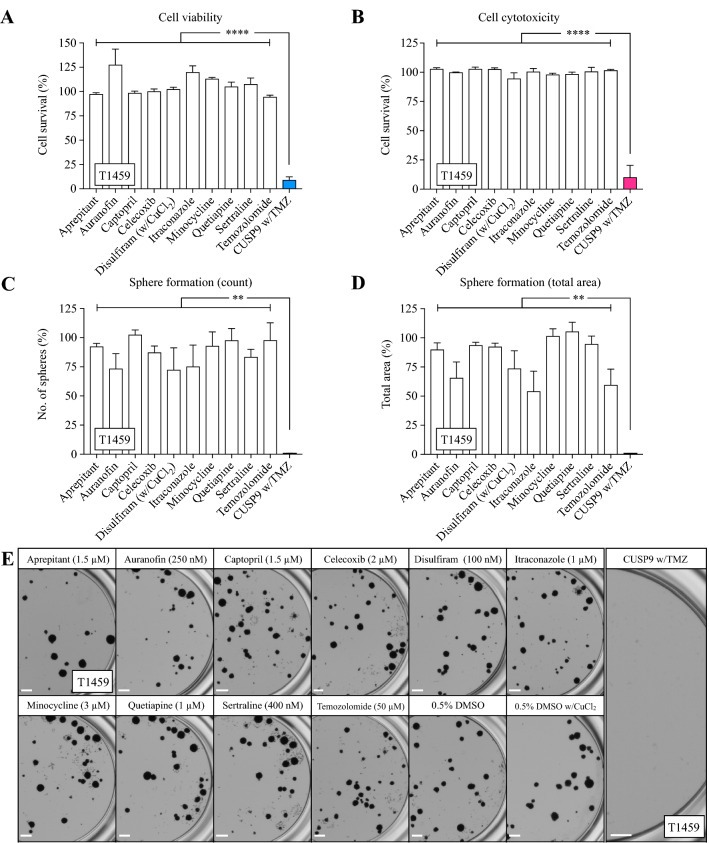
The efficacy of the individual drugs and the CUSP9 combination in T1459. **a**, **b** None of the drugs in the CUSP9 or TMZ reduced the cell survival individually evaluated by the cell viability or cytotoxicity assay; however a significant effect was observed when applied as a drug combination in CPCs (both *p* < 0.0001, one-way ANOVA). **c**–**e** Selected individual effects of drugs in CPCs were found in the sphere-forming assay evaluated by count (AUR, DSF, and ITZ) and total area of spheres (AUR, DSF, ITZ, and TMZ); however, a significant combination effect was observed eradicating all spheres (both *p* < 0.01, one-way ANOVA). **e** Representative images of sphere formation following exposure with indicated drugs. Scale bar 500 µm. Each bar in the graphs represents the mean and standard error to the mean of ≥ 3 individual experiments

We next investigated the effect of all drugs individually along with the combined effect of CUSP9 with TMZ (w/TMZ). In T1459, the drugs were not effective individually; we found, however, a significant combination effect when evaluating both viability and cytotoxicity (both *p* < 0.0001, Fig. 3a, b). We further investigated the efficacy of CUSP9 in a third assay evaluating sphere formation. Despite the limitations of detailed clonal analysis using sphere-forming assays (Singec et al. 2006), marked differences in the number of spheres and sphere diameter capture inhibitory effects. We observed selected individual effects of drugs in reducing the total number of spheres (AUF, DSF, and ITZ, Fig. 3c) and the total area of spheres (AUR, DSF, ITZ, and TMZ, Fig. 3d). However, the CUSP9 w/TMZ combination confirmed a significant combination effect by completely eradicating all spheres (both *p* < 0.01, Fig. 3c–e).

**Fig. 4:**
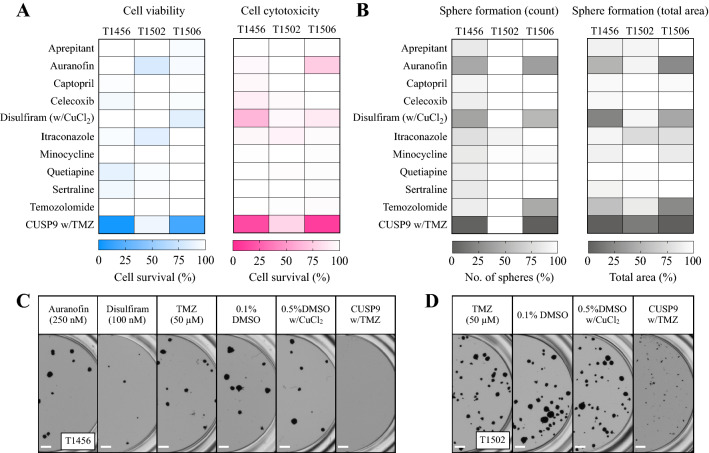
The efficacy of individual drugs and CUSP9 combination in primary GSC cultures. **a** Heat maps (blue; viability, red; cytotoxicity) of the individual drug responses and the CUSP9 combination in three primary GSC cultures display the combination effect of the combination in T1456 and T1506 and the resistance in T1502. **b** Heat maps of the individual drug responses and the CUSP9 combination in the sphere-forming assay display the similar combined effect in T1456 and T1506, while T1502 was sensitive evaluated by the total area of spheres (*p* < 0.001, one-way ANOVA). The cytotoxicity evaluation of T1456 represents the mean of two individual experiments; all other values represent the mean of  ≥ 3 individual experiments in every tumor. **c**, **d** Representative images of sphere formation in T1456 and T1502, respectively, after exposure to indicated drugs. In T1456, a substantial effect of DSF at 100 nM was found; however, the CUSP9 w/TMZ confirmed the combination effect completely eradicating all spheres. Scale bar 500 µm

We further investigated the effect of all drugs individually and in the CUSP9 w/TMZ combination in three additional primary GSC cultures (T1456, T1502, and T1506). In two of the cultures (T1456 and T1506) the same effect was found across all assays (Fig. 4a–c). In one culture (T1502), however, the combination had very limited efficacy (> 75% cell survival) in both the viability and cytotoxicity evaluation (Fig. 4a). Furthermore, in T1502, the combination treatment did not reduce the total number of spheres, but inhibited the capacity to form large spheres (*p* < 0.001, Fig. 4b, d).

### The efficacy of CUSP9 in a heterogeneous population of GSC cultures

**Fig. 5:**
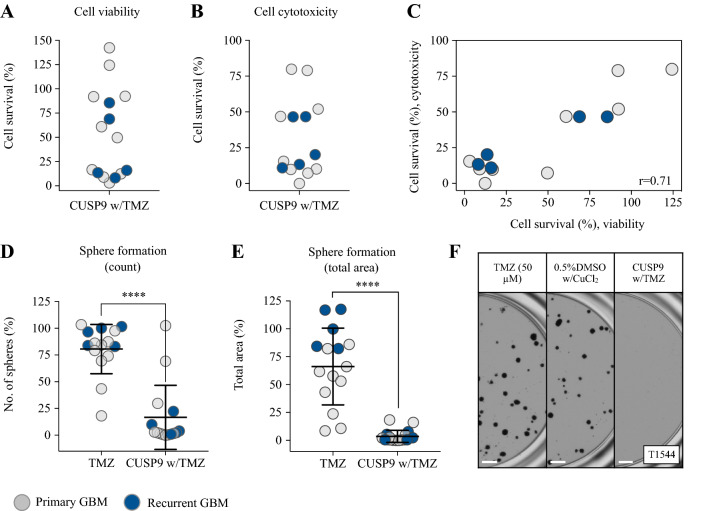
The efficacy of CUSP9 combination in a heterogeneous GSC culture population. **a**, **b** The efficacy of CUSP9 w/TMZ in up to a total of 15 GSC cultures from both primary and recurrent GBM evaluated by viability and cytotoxicity, respectively. **c** Ranked correlation (spearman) of sensitivity to CUSP9 from the viability and cytotoxicity evaluation. **d**, **e** The efficacy of the CUSP9 combination in all GSC cultures compared to TMZ monotherapy (*p* < 0.0001, paired sample *t* test). **f** Representative images of sphere formation in the recurrent GSC culture T1544 display the resistance to TMZ and sensitivity to CUSP9. Scale bar 500 µm

The CUSP9 approach was originally coined for the treatment of recurrent GBM (recGBM) (Kast et al. 2013, 2014). We, therefore, cultured GSCs from five patients undergoing surgery for relapsed disease to evaluate efficacy of the CUSP9 w/TMZ combination in recGBM. As GBM display complex tumor heterogeneity, we further included additionally six primary GSC cultures, adding up to 15 individual GSC cultures in total (Fig. 5). The viability and cytotoxicity evaluation demonstrated a continuum in response patterns ranging from insensitive (> 75% cell survival) to highly sensitive (< 25% cell survival), with seven and eight clustering as highly sensitive, respectively, which included both primary and recurrent GSC cultures (Fig. 5a, b). The correlation of drug responses displayed an excellent correlation (*p* < 0.01, Fig. 5c). The longer incubation for evaluation of sphere formation resulted in more cultures categorized as highly sensitive, and evaluation by the total area of spheres suggested a broad effect. To compare efficacy of the combination treatment to the standard-of-care (TMZ), we compared the results from the sphere-forming assay, as incubation > 72 h in required for detecting TMZ efficacy at CPCs (Lun et al. 2016). The combination regimen displayed a significant superior efficacy to TMZ monotherapy (*p* < 0.0001, Fig. 5d, e). Interestingly, all GSC cultures from recGBM were highly resistant to TMZ monotherapy compared to the heterogeneous response in primary GSC cultures (Fig. 5d–f).

### CUSP9 responsiveness cultures are enriched for proneural subtype

In the 14 cultures tested for sensitivity to the CUSP9 combination w/TMZ evaluated by the viability and cytotoxicity assay [one culture (T1561) failed the cytotoxicity evaluation], both primary and recurrent GSC cultures were found ranging from less to highly sensitive. However, 50% of the cultures (*n* = 7) clustered with a highly sensitive response pattern (< 25% cell survival in both assays) (Fig. 5c). We explored possible patient- or GSC culture-specific traits associated with high response. We found that all high responders were of proneural subtype; however, cultures of proneural subtype were also among the insensitive cultures (Online Resource 1). Correlation of response patterns to CD133 expression, proliferative capacity, MGMT-promoter methylation, and patient age or survival did not establish any significant relationships.

### CUSP9 reduces cancer stem cell signaling pathway activity

**Fig. 6:**
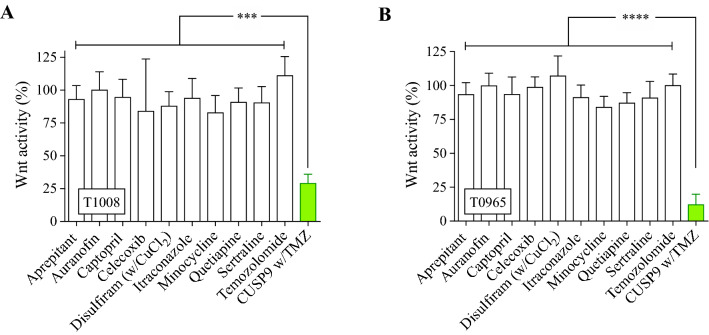
Inhibition of Wnt-signaling after exposure to CUSP9 w/TMZ. **a**, **b** In the luciferase assay, the exposure to CUSP9 combination displayed a significant inhibition of Wnt-activity in T1008 (*p* < 0.001, one-way ANOVA) and T0965 (*p* < 0.0001, one-way ANOVA), respectively. Each bar represents the mean and standard error to the mean of  ≥ 3 individual experiments

The rationale of the drug composition in CUSP9 was to add drugs that both inhibit general growth factor-related signaling pathways (e.g., Akt, mTOR, and STAT), and specifically target the stem cell population in GBM (e.g., ALDH and hedgehog signaling) (Kast et al. 2013, 2014). Although not outlined in the original CUSP9 protocol; we noticed that two of the drugs (celecoxib, quetiapine) also have been reported to attenuate Wnt-signaling in commercially available GBM cell lines (Sareddy et al. 2013; Wang et al. 2017). Previously reporting on the importance of dysregulated Wnt-signaling in GSCs (Sandberg et al. 2013; Kierulf-Vieira et al. 2016), we investigated whether Wnt-signaling could play a role in the CUSP9 protocol. We, thus, explored both the individual drugs and the complete combination for functional inhibition of canonical Wnt-activity and found a significant reduction in Wnt-activity by the combined treatment (*p* < 0.001, Fig. 6a, b). This attenuation of the Wnt-activity was a combination effect, as we could not ascribe the inhibition to any drugs individually, including no substantial attenuation by celecoxib or quetiapine at CPCs.

## Discussion

Using clinically achievable concentrations, we provide, in this study, experimental data of a functional combination effect utilizing a coordinated pharmacological blockade by nine well-known drugs approved for non-oncological indications together with TMZ (the CUSP9 strategy) in patient-derived GSC cultures from both primary and recurrent GBMs.

As some GBM patients already supplement the conventional treatment with the CUSP9 strategy, we conducted this study with a clinical focus. However, mirroring clinical practice in preclinical studies is challenging. One fundamental aspect is the determination of drug concentrations that are achievable within the tumor of the patients (Liston and Davis 2017). What drug concentrations to pursue preclinically to reflect a clinical situation are not well defined (Smith and Houghton 2013). It has been suggested that preclinical drug levels can be decided using *C*_max_ as an upper reference limit to mirror a clinical situation and remove potential off-target effects of individual drugs (Liston and Davis 2017). In this study, we initially determined the dose–response relationships to each drug spanning the therapeutic range to investigate the inhibitory effects of individual drugs in clinically achievable concentrations. This led us to more carefully investigate a combined effect by reducing dominant effects of single drugs in CPCs. However, CPCs and *C*_max_ varies by different dosing schedules and routes of administration (Liston and Davis 2017). In this study, we decided to pursue concentrations in the lower end of reported clinical values at standard dosing regimens for the individual drug intended use. The adaptation of our experimental conditions to clinical plasma concentrations may, however, not reflect therapeutic drug concentrations achievable intratumorally or within the brain parenchyma. Although the drugs in CUSP9 are designed based on the properties of the drugs to cross the blood–brain barrier (Kast et al. 2014), penetrability and brain tissue levels of the individual CUSP9 drugs are unclear, as are intracerebral concentrations of most anticancer agents (Pitz et al. 2011). Moreover, when interpreting the results on combined effects, it is important to consider the limitations of the artificial stable drug exposure in vitro, which do not reflect the complex pharmacokinetics in patients–a complexity that increases by orders of magnitude when adding up to ten drugs in combination in vivo.

Different assays capturing different evaluations of cell death create more robust data when reporting drug efficacy in preclinical studies (Begley and Ellis 2012). In this study, the evaluation of overall efficacy after exposure to CUSP9 w/TMZ using cell viability and cytotoxicity readouts displayed a very good correlation. In selected drug responses (e.g., AUR and ITZ, Fig. 1), the tetrazolium-based cell viability assay displayed a bimodal dose–response pattern with a paradoxical increase in cell viability before the inhibitory effects occurred. This effect was similarly found for the same drugs when tested in CPCs (Fig. 2a). This may suggest a growth stimulatory effect; however, the inhibitory effects of these drugs evaluated by the sphere-forming assay (Fig. 2c, d) suggest that the response rather reflected a cellular stress response increasing the metabolic activity of the cells. A cellular stress response is biased in cell-based assays where metabolic activity is used as a surrogate marker for cell viability. This observation is in accordance with reported inaccuracies in using tetrazolium-based cell viability assays when interpreting subtle differences in cell viability evaluations (Sims and Plattner 2009; Stepanenko and Dmitrenko 2015). It further points to the importance of using different readouts for more accurate evaluation of drug efficacy in preclinical studies. For the sphere-forming assay, cells were incubated for a longer time. This could explain some of the more pronounced effects that were observed in this experimental setup.

Although not described as a key target of the CUSP9 combination, we found a significant reduction of Wnt-signaling activity by CUSP9 w/TMZ treatment, suggesting that this pathway may play a role in the combined treatment effect. In concentrations tenfold higher than clinically achievable in commercially available GBM cell lines, celecoxib and quetiapine have been shown as inhibitors of canonical Wnt-signaling in GBM (Sareddy et al. 2013; Wang et al. 2017). In this study, using CPCs, we found no individual effects of the drugs comprised in the CUSP9, and thus, the inhibition of the signaling pathway was related to a combined effect, which suggests a significance of the Wnt-signaling pathway as a mediator of the combined effect of CUSP9 w/TMZ. This finding, however, requires further studies exploring both the heterogeneity between patient-derived GSC cultures and exploring the entire spectra of expected key signaling target pathways.

In this study, the efficacy of the CUSP9 combination was evaluated using patient-derived GSC cultures from both primary and recurrent GBMs. Compared to commercially available GBM cell lines grown in serum, the GSC model system is recognized as a superior representation of GBM as it can recapitulate the cellular spectrum and malignant phenotype of GBM upon xenotransplantation, and preserve genomic feature of the parent tumor (Lee et al. 2006; Vik-Mo et al. 2010; Davis et al. 2016; Rosenberg et al. 2017; Lan et al. 2017). Experimental models that faithfully recapitulate the human disease are essential for preclinical studies; however, we acknowledge that selection of the aggressive GSC population underestimates the complexity in drug responses compared to the situation in vivo. Interestingly, we found that the recGBM cultures displayed resistance to TMZ, consistent with cultures being derived from recurrent tumors after TMZ treatment. This finding supports the external validity of the presented drug sensitivity data. Despite demonstrating a combination effect, we have not delineated whether all, some, or only a few of the drugs are required for the observed effect. However, patients following the CUSP9 strategy aim to utilize a combination of all drugs; therefore, detailed elucidation of whether only some of the drugs are required for the observed combination effect was not the scope of the current investigation. The use of patient-derived cultures from both treatment-naïve and pretreated tumors suggests that the combined effect can be found in several cultures sampled from a genetically heterogeneous GBM population. And as in vitro sensitivity to the standard-of-care, TMZ, a GSC gene signature, and the ability of GSC to expand as tumorspheres are independent predictors of patient outcome (Laks et al. 2009; Sandberg et al. 2013; D’Alessandris et al. 2017), a growing body of experimental data suggests the clinical relevance of using the GSC model system in preclinical GBM research.

In summary, using clinically achievable drug concentrations, we have added preclinical experimental data of a combined effect utilizing the CUSP9 strategy with TMZ in patient-derived GSCs, which supports clinical assessment of this approach. However, predicting response in individual cultures will require further profiling of GSCs. As some patients adhere to the CUSP9 treatment strategy outside of clinical trials within a do-it-yourself approach, we emphasize the importance of providing experimental data and trials with systematic follow-up of new treatment approaches consisting of drugs available for patients outside of specialized neuro-oncology treatment centers for adequate delineation of efficacy and toxicity.

## Electronic supplementary material

Below is the link to the electronic supplementary material.

Patient and GSC culture characteristics (XLSX 40 kb)

## Data Availability

The data sets generated during and analyzed during the current study are available from the corresponding author on reasonable request.
